# The use of angiotensin receptor blockers in dementia prevention

**DOI:** 10.1590/1980-5764-DN-2023-3006

**Published:** 2023-09-04

**Authors:** Jordana de Araujo Müller, Laura Jacques Giacobe, Vanise Grassi, André Luiz Rodrigues Palmeira

**Affiliations:** 1 Universidade do Vale do Taquari, Centro de Ciências Médicas, Lajeado RS, Brazil.

**Keywords:** Dementia, Alzheimer Disease, Hypertension, Angiotensin Receptor Antagonists, Demência, Doença de Alzheimer, Hipertensão, Antagonistas de Receptores de Angiotensina

## Abstract

Alzheimer’s disease (AD) and dementia are preventable and highly prevalent diseases, as is systemic arterial hypertension. Thus, it is speculated that angiotensin receptor blockers (ARBs) may be neuroprotective against AD.

**Objective:**

The aim of this study was to evaluate if the use of ARBs confers a neuroprotective effect on AD, through a systematic review.

**Methods:**

Studies published on Embase, LILACS, SciELO, and PubMed were evaluated. The selection of the studies included those that evaluated the use of antihypertensive drugs in individuals with a previous diagnosis of mild cognitive impairment. The data were extracted with the Cochrane Effective Practice and Organization of Care (EPOC) form. The risk of bias was evaluated by the EPOC “Risk of bias tool.”

**Results:**

A total of 12 articles were identified, and 3 articles were selected. Two of them analyzed the use of ARB/ACEI versus other antihypertensives and the development of dementia.

**Conclusion:**

There is a tendency for ARBs to be superior to other antihypertensives in preventing dementia.

## RESUMO

A doença de Alzheimer (DA) e a demência são doenças potencialmente preveníveis, assim como a hipertensão arterial sistêmica. Dessa forma, especula-se que os bloqueadores dos receptores de angiotensina (BRA) tenham efeito neuroprotetor contra a DA.

**Objetivo:**

Avaliar se o uso de BRA confere efeito neuroprotetor para DA, por meio de uma revisão sistemática.

**Métodos:**

Foram avaliados estudos publicados nas plataformas Embase, Literatura Latino-Americana e do Caribe em Ciências da Saúde (Lilacs), Scientific Electronic Library Online (SciELO) e United States National Library of Medicine (PubMed). Os estudos incluídos avaliaram o uso de anti-hipertensivos em indivíduos com diagnóstico prévio de comprometimento cognitivo leve. Os dados foram extraídos com base no formulário da EPOC. Risco de viés foi avaliado por meio da ferramenta da Cochrane Effective Practice and Organisation of Care (EPOC) “Risk of bias tool”.

**Resultados:**

Foram encontrados 12 artigos e três foram selecionados. Dois analisaram o uso de BRA/IECA *vs*. o uso de outros anti-hipertensivos e o desenvolvimento de demência.

**Conclusão:**

Há uma tendência de que os BRA sejam superiores a outros anti-hipertensivos na prevenção da demência.

## INTRODUCTION

According to the World Health Organization (WHO), it is estimated that 35.6 million people worldwide have a diagnosis of Alzheimer’s disease (AD). It is expected that this prevalence will double by the year 2030 and triple by 2050^1^. Concomitantly, the report The World Population Prospects 2019, by the United Nations (UN), highlights that the world population is undergoing an aging process, due to increased life expectancy and declining fertility rates, and by the year 2050, it is projected that 16% of the world’s population will be over 65 years old^2^. AD, which accounts for about 50–60% of all dementia cases^3^, will be a major challenge to global health, with increasing prevalence and large financial impact, especially in developing countries^4^.

AD courses with a clinical picture that changes and worsens as the disease progresses, composed of cognitive symptoms such as memory loss, language disorders, attention and executive function disorders, temporal/spatial disorientation, apraxia, perceptual and visual disorders-spatial, and anosognosia, as well as neuropsychiatric symptoms that frequently precede cognitive symptoms and are often treated pharmacologically without suspicion of AD^3^. In advanced stages, cognitive disorders and therefore functional disability are established in such a way that the affected individual is bedridden, unable to perform self-care activities, and with great demand for assistance^3^. Several pathophysiological mechanisms are associated with this disease, and its understanding is essential to face this problem.

The main histopathological feature associated with AD is the aggregation of beta-amyloid 42, resulting in amyloid plaques, and hyperphosphorylated tau protein, resulting in neurofibrillary tangle formation^3^. It is a subject of study what triggers these pathological features. However, there is evidence of an interaction between genetic and epigenetic factors, which characterizes AD as a complex disease. In this sense, changes in neurotransmitters, their metabolism enzymes, and their receptors are a consequence of this phenomenon. Furthermore, AD shares characteristics with vascular dementia^3^. It is seen that, among the main risk factors for AD, arterial hypertension is included as an important vascular factor^3^. Systolic blood pressure (SBP) control in middle age should aim for 130 mmHg or less to delay or prevent dementia^5^.

Compared to the brains of normotensive individuals, individuals with a history of hypertension show increased amyloid burden, brain atrophy, and neurofibrillary tangles formation. Consistent with these findings, it is noteworthy that individuals with abnormal plasma levels of amyloid protein and elevated BP in middle age are at an especially high risk of developing AD in the future^6^. Moreover, the renin-angiotensin system (RAS) plays a fundamental role in the regulation of BP, that is, it indirectly influences the pathogenesis of AD. Nevertheless, RAS also acts directly in the pathophysiology of AD, through the action of its components in the underlying histopathological events of the disease^6^.

It is therefore believed that angiotensin receptor blockers (ARBs) exert a neuroprotective effect on AD. ARBs increase the expression of ART2 and ART4, which elevates levels of the angiotensin-converting enzyme. This degrades the beta-amyloid proteins that accumulate and generate DA, therefore exerting neuroprotection^7^. Thus, it is important to focus not only on therapeutics in symptomatic treatment, but also on the approach of modifiable risk factors, with the objective of generating a drop in future statistics, reducing the impact of AD, and promoting quality of life.

In this sense, the present study analyzed the action of antihypertensive drugs in the prevention of AD. Several studies indicate that ARBs are superior to beta-blockers, diuretics, and ACE inhibitors in preventing cognitive decline^6^.

This study aimed to evaluate if the use of ARBs confers a neuroprotective effect on AD. Specific objectives are as follows:

- To compare the AD mortality among hypertensive individuals using ARBs and other antihypertensive drugs.
- To investigate if the use of ARBs reduces the incidence of AD in cognitively healthy hypertensive individuals or with minor neurocognitive disorder.
- To estimate if the use of ARBs reduces the speed of conversion of cognitively healthy hypertensive individuals or those with minor neurocognitive disorder to AD.
- To assess if the use of ARBs can prevent AD in cognitively healthy hypertensive patients or those with minor neurocognitive disorder.

## METHODS

This study is a systematic literature review based on qualitative analysis of publications and its synthesis in a table with main findings. 

### Database and research

The electronic databases of Embase, PubMed, and LILACS were searched. The Emtree terms were used in Embase research included “*dementia*” AND “*high blood pressure*.” For PubMed, the following MeSH (Medical Subjective Headings) terms were used: “*mild cognitive impairment*” AND “*dementia*” AND “*high blood pressure*.” The research included all relevant studies published before August 29, 2021.

### Study selection

The considered studies were clinical trials and cohort prospective and retrospective. Furthermore, they needed to meet the following criteria:

- The population consisted of adults older than 40 years;
- Both sexes;
- No nationality restriction;
- A previous diagnosis of high BP before the outcome;
- On regular antihypertensive treatment; and
- Cognitively healthy or with a diagnosis of mild cognitive impairment (MCI) at the beginning of the study (according to DSM-IV).

The expected intervention was the use of antihypertensive drugs.

The outcomes considered were those that presented the incidence of dementia (according to DSM-IV). In addition, it evaluated the specific incidence of AD and the baseline change in the mini-mental state examination (MMSE) performance.

Two reviewers (JAM and LJG) independently screened the studies based on their titles and abstracts, evaluating the studies eligibility. After this first step, the remaining studies were fully analyzed for eligibility according to the established criteria. In cases of disagreement regarding the eligibility of the study in any step, the other two reviewers (ALRP and VG) were consulted.

### Data extraction

Two reviewers (JAM and LJG) extracted the following data independently and in duplicate, using the standardized form from the Cochrane Effective Practice and Organization of Care (EPOC). Relevant information was collected about the study design, participants (number and epidemiologic/demographic characteristics), and a full description of the realized intervention. Outcomes details were also extracted (including a description of how and when the outcomes were measured).

### Risk of bias

The EPOC “Risk of bias tool” was used to evaluate the risk of bias in all the studies. This tool includes five domains of bias: selection bias, performance bias, detection bias, attrition bias, and reporting bias. Two reviewers (JAM and LJG) evaluated the risk of bias in duplicate and independently, and when disagreement happened, a third reviewer (VG and ALRP) was consulted to resolve the issue.

## RESULTS

### Database research

**Figure 1.**
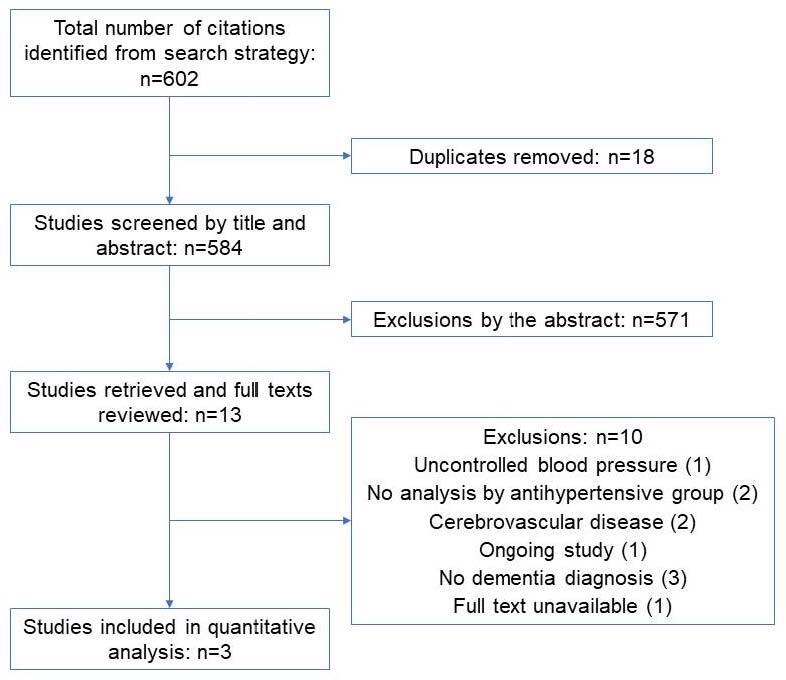
Flowchart for publication selection.

A total of 12 articles were identified, but only 3 articles were selected for a detailed evaluation based on the complete reading of the articles and the eligibility criteria. After this analysis, three articles were included in the systematic review. A flowchart of the research result is shown in Figure 1.

### Studies and population characteristics

Among the three selected articles for this review, Wharton et al.^8^, selected a lot of Caucasians. Peters et al.^9^ recruited the participants from Eastern European countries and from China. Wharton et al.^10^, used data from the Rush Alzheimer’s Disease Core Center (USA). All of the participants in the three articles were hypertensive and were taking antihypertensive studies. The mean age was between 70 and 80 years. 

The studies approached adults (over 18 years) of both sexes without a previous diagnosis of dementia.

Table 1 presents the characteristics of the studies.

**Table 1.** Characteristics of the studies.

Article	Population	Intervention	Outcomes	Conclusion
Wharton et al.^8^	83 individuals with MCI who were taking an antihypertensive at baseline. 38 RAS users. Age: 82.6±7.2 years. Sex: 45% male. Black: 13.9%. 45 RAS non-users. Age: 83.7±5.9 years. Sex: 20% male. Black: 4.4%.	38 participants were allocated in the RAS (renin-angiotensin system) users. They received ACEI (angiotensin converting enzyme inhibitors) or ARB (angiotensin receptor blockers). 45 participants were allocated in the non-RAS users. They received beta blocker, diuretics or calcium channel blockers. Both groups were accompanied for 4 years.	Progression from MCI to AD (Alzheimer’s disease: RAS users=3 (7.9%). Non-RAS users=11 (24.4%). Neuropathological changes: Absolute numbers of plaques in hippocampus CA1 and entorhinal cortex were significantly lower in non-RAS-AHM group. In the fully adjusted model, RAS users had significantly fewer overall neurofibrillary tangles (NFTs) (p<0.01) and fewer tangles in all prespecified regions of interest including the hippocampal CA1 region (p<0.01), the entorhinal cortex (p<0.01), and the average number of NFT in brain regions, including the angular gyrus, inferior temporal, mid-frontal cortex, and superior frontal (p=0.06). RAS users also had higher brain weight (p=0.03) and fewer diffuse plaques (p=0.02). RAS-AHM and non-RAS-AHM users did not differ on neuropathological indices of NIA Reagan, amyloid burden, arteriolosclerosis, Lewy body disease, chronic, micro, or gross infarcts, Braak, or CERAD scores.	The association between use of RAS-AHM and conversion to AD remains significant (p=0.0285). RAS users had significantly fewer overall NFTs (p<0.01) and fewer tangles in all prespecified regions of interest.
Wharton et al.^10^	RAS acting: 488 (312 were taking BBB-crossing medications and 124 were taking non-BBB-crossing formulations). African-American: 90; Caucasians: 398. Age: 74.6±8 years. Male: 46%. Non RAS: 296 (113 were taking a calcium channel blocker, 174 a beta-blocker, and 108 a diuretic). African-American: 117; Caucasians: 552. Age: 75.5±8.8 years. Male: 49%.	488 were allocated in the RAS-acting group, taking ACEI or ARB. 296 were allocated in the non RAS group, taking calcium channel blockers, diuretics, or beta-blockers. Both groups had a follow-up of 3 years.	Conversion rates to AD as a function of RAS-acting medication use: 280 of the 784 participants taking an antihypertensive medication converted to AD. 161 were RAS-acting medication users (33.0%), 119 non-users (40.2%), 98 centrally acting medication users (30.7%), and 48 non-centrally acting medication users (40.0%) converted to AD.	Participants taking BBB-crossing RAS-acting medications would have greater cognitive protection than those taking non-centrally acting medications. Results regarding the rate of decline showed beneficial cognitive and functional effects on the Boston Naming Test (p<0.01), MMSE (p<0.01), and Clinical Dementia Rating-Sum of Boxes (CDR-SOB p<0.01).
Peters et al.^9^	Participants were recruited from Eastern European countries and from China. 60% woman. Age 83.5 years (3.1). Active group n=1,623. Placebo group n=1,580.	Placebo-controlled trial comparing indapamide SR 1.5 mg or matching placebo with the optional addition of 2 or 4 mg perindopril, or matching placebo to reach a goal BP of less than 150/80 mmHg.	There were 263 cases of incident dementia (3.8/100 patient-years in the placebo compared with 3.3/100 in the actively treated group). Placebo: For SBP and DBP, there was no significant relationship with later dementia (p=0.13 and p=0.43, respectively). For PP, higher PP was associated with increased risk (p=0.032). Intervention: There was no significant relationship between SBP and later dementia (p=0.36). Diastolic pressure showed a clearer and statistically significant U-shaped relationship with later dementia with a calculated nadir of 87.5 mmHg (p=0.0195). Higher PP was associated with increased risk (p=0.0046).	The HYVET trial also collected data relating to incident dementia and found no difference between the active and placebo groups with regard to this endpoint, although there were 8% fewer cases in the actively treated group. That SBP shows no clear relationship with dementia (…)

### Outcomes

Wharton et al.^10^, analyzed a cohort study of 83 individuals with MCI, which represents an intermediate state between the changes of normal aging and dementia^11^, who were treated with an antihypertensive at baseline. Of these, 38 participants were allocated to the RAS group, who received an antihypertensive that acts on the RAS such as angiotensin converting enzyme (ACE) inhibitors or ARBs, and 45 participants were allocated to the non-RAS group, who received antihypertensives that did not act on the RAS such as beta-blockers, diuretics, and calcium channel blockers.

The incidence of progression to AD is significant (p=0.0285), and it was 7.9% in the RAS users and 24.4% in the non-RAS users. Wharton et al.^10^, also analyzed *postmortem* neuropathological changes. The RAS users had significantly fewer overall neurofibrillary tangles (p<0.01) and fewer tangles in all regions of interest, such as the hippocampal CA1 region and the entorhinal cortex.

In addition to the already recognized association between hypertension in middle age and AD, it is essential to elucidate the more specific influence of the RAS — essential in BP regulation — both in the pathogenesis of AD and in the cognitive impairment more broadly, given that the types of dementia share the same imbroglio of lack of therapeutic options.

Wharton et al.^8^, analyzed a cohort of 784 individuals with MCI. In this study, 488 participants were RAS users, constituted for ACE inhibitors (ACE-I) and ARBs drugs, and 296 were non-RAS users, constituted for beta-blockers, calcium channel blockers, and diuretics drugs. Unadjusted conversion rates for each group were as follows: RAS users 161 (33.0%) and RAS non-users 119 (40.2%). Adjusted results show that the conversion rate from MCI to AD was significantly lower for RAS users versus non-users (p=0.04). An additional analysis of the conversion rate from AD to MCI was performed among RAS users of drugs that cross the blood-brain barrier (BBB), therefore showing more action in the central nervous system, and SRA users that do not have a central action. Unadjusted conversion rates for centrally acting RAS users were 98 (30.7%) and for non-centrally acting RAS users were 48 (40.0%). Adjusted results show that the conversion rate from MCI to AD was lower among RAS users — those using centrally acting medication converted less often than non-centrally acting users (p=0.06). 

Wharton et al.^8^ and Peter et al.,^9^, specifically analyzed the results of a placebo-controlled trial comparing indapamide SR 1.5 mg or matching placebo with the optional addition of 2 or 4 mg perindopril or matching placebo to reach a goal BP of less than 150/80 mmHg. The trial found that antihypertensive treatment reduced the risk of total mortality, stroke, and cardiovascular events in those aged 80 years and over with hypertension. Moreover, the trial also collected data relating to incident dementia and found no difference between the active and placebo groups with regard to this endpoint, although there were 8% fewer cases in the actively treated group. In relation to the placebo-treated group, SBP and diastolic BP (DBP) did not show a significant relationship with later dementia (p=0.13 and p=0.43, respectively). For pulse pressure (PP), a higher PP was associated with increased risk (p=0.032). In the actively treated group, there was no significant relationship between SBP and later dementia (p=0.36). Higher PP was associated with increased risk (p=0.0046). Interaction terms for BP and treatment group were not statistically significant for systolic (p=0.06), diastolic (p=0.38), or PP (p=0.78), indicating no definite difference in the relationship between BP and dementia by treatment group, although doubt remains for SBP. 

### Risk of bias analyses

**Figure 2.**
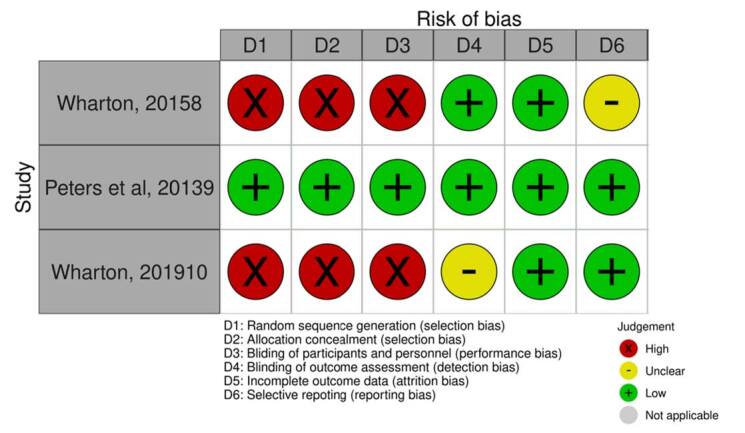
Risk of bias summary. Review authors’ judgement about each risk of bias item for each included study. These items were classified as “adequate” (low risk of bias) with +, “inadequate” (high risk of bias) with -, or “unclear” with?

Risk of bias assessment is presented in Figure 2.

## DISCUSSION

The main features associated with AD are amyloid and hyperphosphorylated tau aggregates, amyloid plaques, and neurofibrillary tangles^12^. Amyloid plaques result from the extracellular accumulation of abnormally folded beta-amyloid protein^12^. Neurofibrillary tangles and hyperphosphorylated tau filaments, especially helical ones, tend to deposit initially in the entorhinal cortex and hippocampus^12^. According to Sperling et al.^13^, three stages are part of the preclinical phase of AD. The first stage has evidence of beta-amyloid accumulation but no detectable evidence of additional brain changes suggestive of neurodegeneration or cognitive loss. In the second stage, there is evidence of amyloid positivity and the presence of one or more markers of AD-related neuronal injury, and in the third stage, there is amyloid accumulation, early neurodegeneration, and evidence of subtle cognitive decline, therefore approaching the clinical criteria for MCI. This pathophysiological cascade may start years, if not decades, before the onset of clinical dementia, which courses with more advanced lesions, characterized by an accumulation of neurodegeneration^13^.

There are many risk factors associated with the development of dementia, with high BP (HBP) being one of the most significant because of its high prevalence and various treatment options that are modifiable without the expense of great resources^6^. HBP interrupts the process of brain autoregulation and leads to chronic oligemia^6^. The result of this reduction in perfusion is endothelial dysfunction, oxidative stress, and other metabolic changes. In addition, oligemia also downregulates important proteins for memory and brain plasticity, as well as upregulating APP, oligomerization of beta-amyloid, and phosphorylation of neuronal tau^6^. In this context, a recently published study shows that controlled BP in hypertensive patients is significantly associated with a lower risk of general dementia, vascular dementia, and AD; above all, the preventive effect of controlled BP for AD tends to be stronger in people over 60 years^14^. Therefore, corroborating our results, early antihypertensive treatment may be a reasonable tool in preventing dementia.

However, contrary to our findings, Ding et al.^15^, found no evidence that a specific class of antihypertensive drugs was more effective than others in reducing the risk of dementia in their meta-analysis performed with six prospective studies, which analyzed the risk of developing dementia in hypertensive patients treated with one of five classes of antihypertensive drugs: angiotensin-converting enzyme inhibitors (ACEIs), angiotensin II receptor antagonists (ARBs), beta blockers, calcium channel blockers (including long- and short-acting preparations), and diuretics. However, again, the results suggest that the use of antihypertensive drugs can effectively reduce the risk of dementia. Despite the large total sample, the study presented some small gaps, such as the low number of cases in the group of ARB users in the HBP strata, making it difficult to estimate class associations. Furthermore, they had little power to investigate specific drugs within a class, which may differ in properties such as those related to the passage of the BBB, aspects that may have generated the divergences with our findings.

Moreover, antihypertensive drugs may have a neuroprotective effect by acting on the RAS. In vitro studies have demonstrated the role of ACE in the degradation of Aβ peptides, interrupting the development of amyloid plaque^16^. This finding suggests that ACE inhibitors may promote βA accumulation in the brain, and it fits with our main hypothesis, which is that ARBs may protect against dementia. According to Regenold et al.^17^, Aβ42 is the predominant form composing plaques within brain tissue. Individuals with AD have a reduced ability to clear Aβ42 from the brain compared to healthy individuals. Regenold et al.^17^, analyzed the levels of Aβ42 in individuals with cognitive impairment taking ACE inhibitors. They found that individuals in treatment with ACE inhibitors had elevated levels of plasmatic Aβ42 in comparison with the group that did not use this medication class (p=0.006).

In this context, there are already readily available and numerous amounts of drugs that act in the RAS and can be used in the therapeutic measures of dementias, especially AD, like ARBs that work through the unopposed action of ANGII on AT2 receptors, because as the AT1 receptor is blocked, these drugs increase the concentration of ANGII to act on the AT2 receptor^16^.

The elements that make up the RAS can be divided into a “classic way” and a “regulatory way,” which tries to counterbalance the previous way^18^. In principle, in the classical RAS pathway, angiotensinogen is converted into angiotensin I (ANGI) by the enzyme renin^18^. Then, ANGI is converted to angiotensin II (ANGII) by ACE. In this way, there are two types of receptors for ANGII: ART1, which is responsible for the vasoconstrictor outcome, and ART2, which antagonizes the function of ART1, causing vasodilation and thus establishing the balance. In view of this, in the context of ART1, several drugs were made to reduce production (renin inhibitors and ACE inhibitors) or signaling mediated by ANGII (ARBs) in order to reduce the vasoconstrictor effect, which also contributes to the treatment of hypertension^18^.

In view of the findings of this study, we conclude that there is some evidence that ARBs have a neuroprotective role for dementia, since patients treated with this class of antihypertensive were among the individuals who had a lower incidence of progression from MCI to AD in the analyzed publications. However, our findings involve groups with other classes of antihypertensives with RAS action, especially drugs that exceed the BBB, which also show signs of a protective effect. Therefore, more specific studies for each class are needed to assess their specificities. It is possible, through our results, to state that the use of ARBs is an accessible and widespread tool with promising neuroprotective potential for dementia.
